# Education Research: Virtual Residency Interviews and the Second Look: The Mayo Clinic Neurology Tri-Site Experience

**DOI:** 10.1212/NE9.0000000000200095

**Published:** 2023-10-16

**Authors:** Elizabeth A. Mauricio, Elizabeth A. Coon, Erika D. Driver-Dunckley, Stephen W. English, Jeremy K. Cutsforth-Gregory, Marie F. Grill, Jennifer M. Martinez-Thompson, Lyell K. Jones, for Mayo Clinic Adult Neurology Residency Program Leadership

**Affiliations:** From the Department of Neurology (E.A.M., S.W.E.), Mayo Clinic, Jacksonville, FL; Department of Neurology (E.A.C., J.K.C.-G., J.M.M.-T., L.K.J.), Mayo Clinic, Rochester, MN; and Department of Neurology (E.D.D.-D., M.F.G.), Mayo Clinic, Scottsdale, AZ.

## Abstract

**Background and Objectives:**

The 2023 Match cycle was the third virtual interview season for adult neurology residency programs. This recruitment cycle differed from years prior because an in-person second-look event was offered by some programs to complement the virtual interview day. We sought feedback from those who interviewed for adult neurology residency positions at Mayo Clinic in Arizona, Florida, and Minnesota to gain applicants' perspectives on such events and identify best practices to improve the interview experience for future applicants.

**Methods:**

Virtual interviews were conducted for adult neurology residency positions at Mayo Clinic in Arizona, Florida, and Minnesota for the 2023 Match. Each site hosted optional, in-person, no-stakes second-look events after program rank lists were finalized. After Match Day, interviewees at all 3 sites were invited to complete an anonymous electronic survey using Qualtrics software to gather feedback on the in-person events.

**Results:**

Of the 240 interviewees for adult neurology residency positions at Mayo Clinic, 52 candidates participated in the in-person second-look event on the Arizona, Florida, or Minnesota campus in 2023. One-third of applicants (80/240) completed the anonymous survey, and of them, 34% (27/80) of respondents had attended a Mayo Clinic second-look event. The desire to step foot on campus, meet the residents in person, and visit the geographic area were the most important reasons driving attendance. Those who did not participate cited financial burden, lack of time, and insufficient notice to make travel plans as the most common factors influencing their decision not to attend. Nearly half of attendees who responded to the survey (10/21, 48%) changed their rank order list after the in-person event. Most of them who participated in a Mayo Clinic second-look event (19/21, 91%) would encourage future applicants to partake.

**Discussion:**

While there are many advantages to virtual residency interviews, most applicants who participated in second-look events found that having an opportunity to visit the program in person was valuable when determining their rank list. Offering an optional, no-stakes, in-person visit allows interviewees to make more informed rank lists. Second-look events have the potential to complement the virtual interview day and may ultimately lead to greater satisfaction for matched applicants.

## Introduction

The coronavirus disease 2019 (COVID-19) pandemic has fundamentally changed the traditional residency recruitment and interview process for both applicants and programs. Virtual interviews have proven valuable in reducing costs, lowering the carbon footprint of interviewees, and improving equity and access for applicants.^1,2^ The virtual space, however, does not always allow an adequate portrayal of a program's culture or surrounding community, which are important factors for neurology applicants when ranking programs.^3,4^

During the 2023 Match cycle, the waning pandemic crisis and easing of travel restrictions opened the door for adult neurology residency programs to host in-person events to complement the virtual interview experience. The primary objective of this study was to assess the potential value of optional, in-person, no-stakes second-look events by sharing feedback from the Mayo Clinic Neurology Residency Program tri-site experience.

## Methods

This systematic investigation was designed with the intent to improve the quality of our resident recruitment and interview process. On this basis, the Mayo Clinic Institutional Review Board deemed the study exempt from formal review and approval.

Residency interviews were conducted virtually for the 3 Adult Neurology Residency Programs at Mayo Clinic in Arizona, Florida, and Minnesota for the 2023 Match. Recruitment Committees at each of the 3 sites finalized their residency program rank order lists before hosting optional, in-person, no-stakes second-look events. Written and verbal communication was provided to applicants by Mayo Clinic School of Graduate Medical Education leadership explicitly stating that participation in these events would not affect ranking of the applicant. Events were held on campus after program rank lists were finalized but before the applicant rank list submission deadline. Centralized oversight of rank lists by Mayo Clinic School of Graduate Medical Education ensured rank lists were not changed after second-look events. Program leadership crafted the unique event itineraries at each of the sites, with all events including campus tours, meals with current residents, and interactions with neurology faculty.

After Match Day, all interviewees for Adult Neurology Residency Programs at Mayo Clinic in Arizona, Florida, and Minnesota were emailed an anonymous electronic survey to gather feedback on the second-look events (eAppendix 1, links.lww.com/NE9/A47). The surveys were sent through the Electronic Residency Application Service platform on March 20, 2023, and survey data collection closed on April 18, 2023, after several reminder emails were sent to interviewees. Qualtrics web-based survey software was used, and the questions were written by the 3 program directors and revised to improve clarity, content, and brevity. The Mayo Clinic Office of Information Security and Privacy has approved Qualtrics as a vendor for distributing, collecting, and storing survey data because the platform meets stringent information security requirements established by our institution.

### Data Availability

Anonymized data not published within this article will be made available upon reasonable request from any qualified investigator.

## Results

A total of 240 applicants were interviewed for adult neurology residency positions at Mayo Clinic in Arizona, Florida, and Minnesota for the 2023 Match. All interviewees were invited to participate in an optional, no-stakes, in-person second-look event at the campus where they interviewed. Fifty-two neurology candidates (22% of interviewees) attended a second-look event at Mayo Clinic. One-third of applicants (80/240) completed the anonymous survey, and of them, 34% (27/80) of respondents had attended a Mayo Clinic second-look event. Of those who completed the survey but did not attend a second-look event hosted by Mayo Clinic, 21% (10/47) attended such an event at another institution.

Of the 46 respondents who did not attend an in-person event, the most significant factors influencing decisions not to attend included lack of time (37%), financial burden (26%), and insufficient notice to make travel plans (11%). Of the 21 survey respondents who attended Mayo Clinic Neurology second-look events, the highest rated reasons to attend included the desire to see the campus in person (57%), desire to visit the geographic area of the program (24%), and the desire to meet the residents in person (14%). The desire to meet the faculty and program director in person were the least influential factors leading to participation. The campus tour (62%), dinner with the residents (33%), and meeting the residents in person (5%) were deemed to be the most valuable aspects of the second-look events. Participation in didactics, patient rounds, or grand rounds were considered the least valued aspects of the day.

Of survey respondents who attended Mayo Clinic Neurology second-look events, 29% (6/21) felt that attendance did affect how programs ranked its candidates, while 14% (3/21) were unsure. For those in attendance, nearly half (10/21) reported that this experience changed their rank order list of programs, ranking the Mayo Clinic program either higher (38%) or lower (10%) after the event (Figure).

**Figure:**
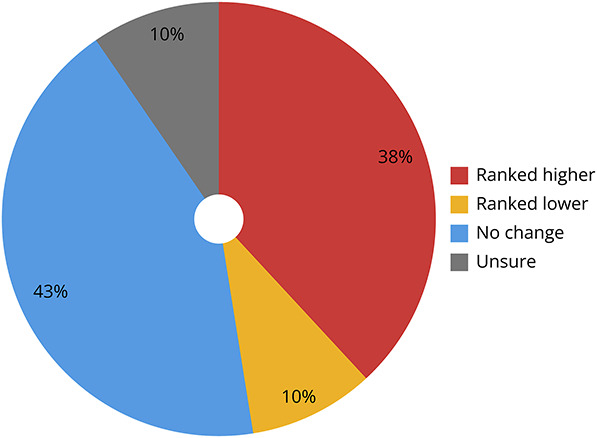
Effect of Second Look on Applicant Rank List Influence of the Mayo Clinic Neurology second-look event on how attendees ranked residency programs in the 2023 Match. 48% of attendees (10/21) changed the order of their rank list after attending the in-person event, while 43% (9/21) did not change their rank list and 10% (2/21) were unsure whether this event influenced their rank list.

When asked what attendees gleaned from the second-look event that they were not able to attain during the virtual interview day, responders commented on the ability to better appreciate the culture and personality of the residents, the interaction between residents and staff, the size and scale of the department and hospital, and the ability to gain greater familiarity with the geographic location (Table). One attendee was seeking, “A gut feeling about fit and ability to visualize myself there.” Of survey respondents who attended a Mayo Clinic Neurology second-look event, 19/21 (91%) would encourage future applicants to attend such events.

**Table:**
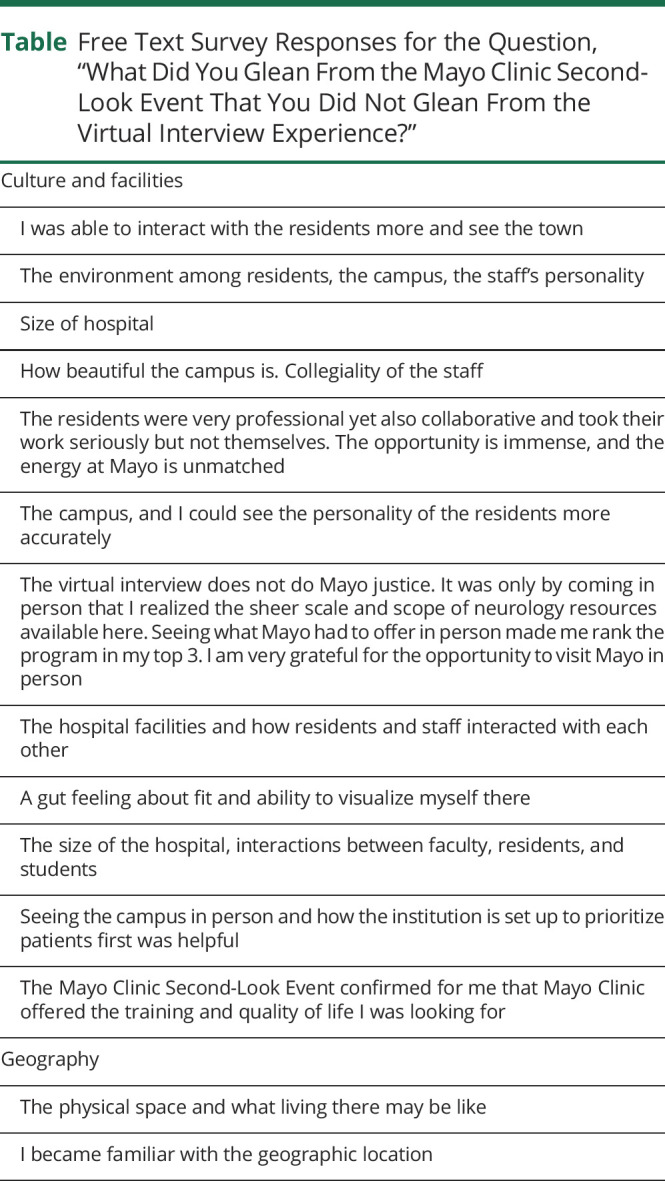
Free Text Survey Responses for the Question, “What Did You Glean From the Mayo Clinic Second-Look Event That You Did Not Glean From the Virtual Interview Experience?”

Culture and facilities
I was able to interact with the residents more and see the town
The environment among residents, the campus, the staff's personality
Size of hospital
How beautiful the campus is. Collegiality of the staff
The residents were very professional yet also collaborative and took their work seriously but not themselves. The opportunity is immense, and the energy at Mayo is unmatched
The campus, and I could see the personality of the residents more accurately
The virtual interview does not do Mayo justice. It was only by coming in person that I realized the sheer scale and scope of neurology resources available here. Seeing what Mayo had to offer in person made me rank the program in my top 3. I am very grateful for the opportunity to visit Mayo in person
The hospital facilities and how residents and staff interacted with each other
A gut feeling about fit and ability to visualize myself there
The size of the hospital, interactions between faculty, residents, and students
Seeing the campus in person and how the institution is set up to prioritize patients first was helpful
The Mayo Clinic Second-Look Event confirmed for me that Mayo Clinic offered the training and quality of life I was looking for
Geography
The physical space and what living there may be like
I became familiar with the geographic location

## Discussion

In the wake of the COVID-19 pandemic, residency programs continue to develop strategies to best construct the ideal interview experience for applicants and programs. Virtual interviews cut travel-related interview expenses for applicants, which historically have amounted to several thousands of dollars, allowing applicants to consider more residency programs.^5^ Of importance, the virtual format has the potential to improve equity and access for less privileged applicants.^2^ Virtual interviews alone can fall short, however, in providing candidates a true understanding of program culture and the local environment and community where they will be spending the next several years.^1,3^ Our results show that second-look attendees find an in-person experience valuable in determining their rank order list. Virtual interviews have not been able to convey important factors for applicants that can be done with in-person events including campus tours, ability to see the geographic area, and interactions with current residents.

The results of the 2022 Neurology Residency Matching Program Applicant Survey illustrated the importance of geographic location and “perceived goodness of fit” for neurology applicants when evaluating programs.^4^ These were the driving factors that led many interviewees to participate in second-look events hosted by Mayo Clinic’s Adult Neurology Residency Programs during the 2023 Match cycle. While participation was not universal among applicants, the most significant barriers included time and money, not disinterest. To provide the most equitable experience possible, programs may help to mitigate these barriers by subsidizing hotel accommodations and meals during the event. In addition, more advanced notice regarding the timing of such events may help applicants plan their schedules accordingly to facilitate participation. The Mayo Clinic sites that defrayed accommodation costs and provided earlier notification experienced greater participation among applicants in their on-campus events. Demographic data were not solicited in the survey to preserve respondent confidentiality, limiting examination of potential disparities affecting attendance at the Arizona, Florida, and Minnesota second-look events.

Assessment of culture and fit is difficult in the virtual space and may drive applicants to embark on self-guided visits if formal events are not offered by programs. While these events may add additional time and financial burden on residency programs, they also offer the opportunity to craft an itinerary highlighting unique program strengths and regional attributes that may not be adequately showcased during a virtual interview day. Most second-look attendees (91%) reported that they would encourage future applicants to participate in such events, which highlights the perceived value from an applicant standpoint. Future studies further examining the value of these events from the perspective of the residency programs would be insightful. By design, the no-stakes second-look visits had no effect on program rank lists.

Despite clear communication and centralized safeguards to ensure that Mayo Clinic Neurology second-look events were optional and would have no impact on ranking of applicants, nearly half of attendees were not confident that this was the case. The visible engagement of program leadership in the second-look activities and the lack of a means for applicants to verify program rank list completion may have contributed to applicant doubt in the no-stakes nature of these visits. Thus, in addition to implementation of measures to ensure visits are truly “no-stakes,” these measures should be transparently and verifiably communicated to future applicants. One proposed solution put forth by the Coalition for Physician Accountability and the Organization of Program Directors Association is the voluntary locking functionality for program rank order lists. This would require programs to certify their rank order lists before applicants and provide a specified time window for applicants to visit programs of interest without concern that such visits can affect program rank lists.^6^

Our results from the Mayo Clinic Neurology Residency experience during the 2023 Match cycle was similar to that of the University of Cincinnati Radiology Residency experience during the 2022 Match cycle. Reasons driving attendance and the perceived benefits were comparable.^7^ Like the University of Cincinnati experience, the Mayo Clinic Neurology in-person second-look visits had a profound impact on attendees. Almost half of Mayo Clinic Neurology second-look participants changed their rank order list after the visit. Helping future adult neurology residents make more informed decisions about where they spend the next 3–4 years of their lives may have significant downstream consequences on program retention and overall educational outcomes. Further studies would be helpful in assessing whether an in-person visit during the interview season influences future resident satisfaction with their matched program and attrition rates and whether adoption of second looks would increase if barriers identified by our survey were mitigated.

While the survey population was from 3 administratively and geographically distinct Mayo Clinic Neurology programs, the results may not be generalizable to all programs and institutions. Demographic data and geographic distance from the hosting institution were not collected in the survey and may have provided additional information on variables affecting participation in the events. The relatively small sample size and low survey response rate are additional limitations that may affect broad generalization of the findings. However, the fact that no survey respondents who attended a Mayo Clinic Neurology second-look event would discourage future applicants from attending illustrates the potential value for interviewees. Further strategies to enhance equity and mitigate barriers to attendance will be important next steps for the education community.

## Appendix Authors

**Table:**

Name	Location	Contribution
Elizabeth A. Mauricio, MD	Department of Neurology, Mayo Clinic, Jacksonville, FL	Major role in the acquisition of data; study concept or design; and analysis or interpretation of data
Elizabeth A. Coon, MD	Department of Neurology, Mayo Clinic, Rochester, MN	Drafting/revision of the article for content, including medical writing for content; major role in the acquisition of data, study concept or design; and analysis or interpretation of data
Erika D. Driver-Dunckley, MD	Department of Neurology, Mayo Clinic, Scottsdale, AZ	Major role in the acquisition of data; study concept or design; and analysis or interpretation of data
Stephen W. English, Jr., MD, MBA	Department of Neurology, Mayo Clinic, Jacksonville, FL	Drafting/revision of the article for content, including medical writing for content
Jeremy K. Cutsforth-Gregory, MD	Department of Neurology, Mayo Clinic, Rochester, MN	Drafting/revision of the article for content, including medical writing for content
Marie F. Grill, MD	Department of Neurology, Mayo Clinic, Scottsdale, AZ	Drafting/revision of the article for content, including medical writing for content
Jennifer M. Martinez-Thompson, MD	Department of Neurology, Mayo Clinic, Rochester, MN	Drafting/revision of the article for content, including medical writing for content
Lyell K. Jones, MD	Department of Neurology, Mayo Clinic, Rochester, MN	Drafting/revision of the article for content, including medical writing for content

## Glossary

COVID-19: coronavirus disease 2019
